# Supplementary figures and images for: Single-Cell Sequencing Identifies Master Regulators Affected by Panobinostat in Neuroblastoma Cells
Source: Genes (Basel). 2022 Nov 29;13(12):2240. doi: 10.3390/genes13122240 (PMC9778475; doi:10.3390/genes13122240)

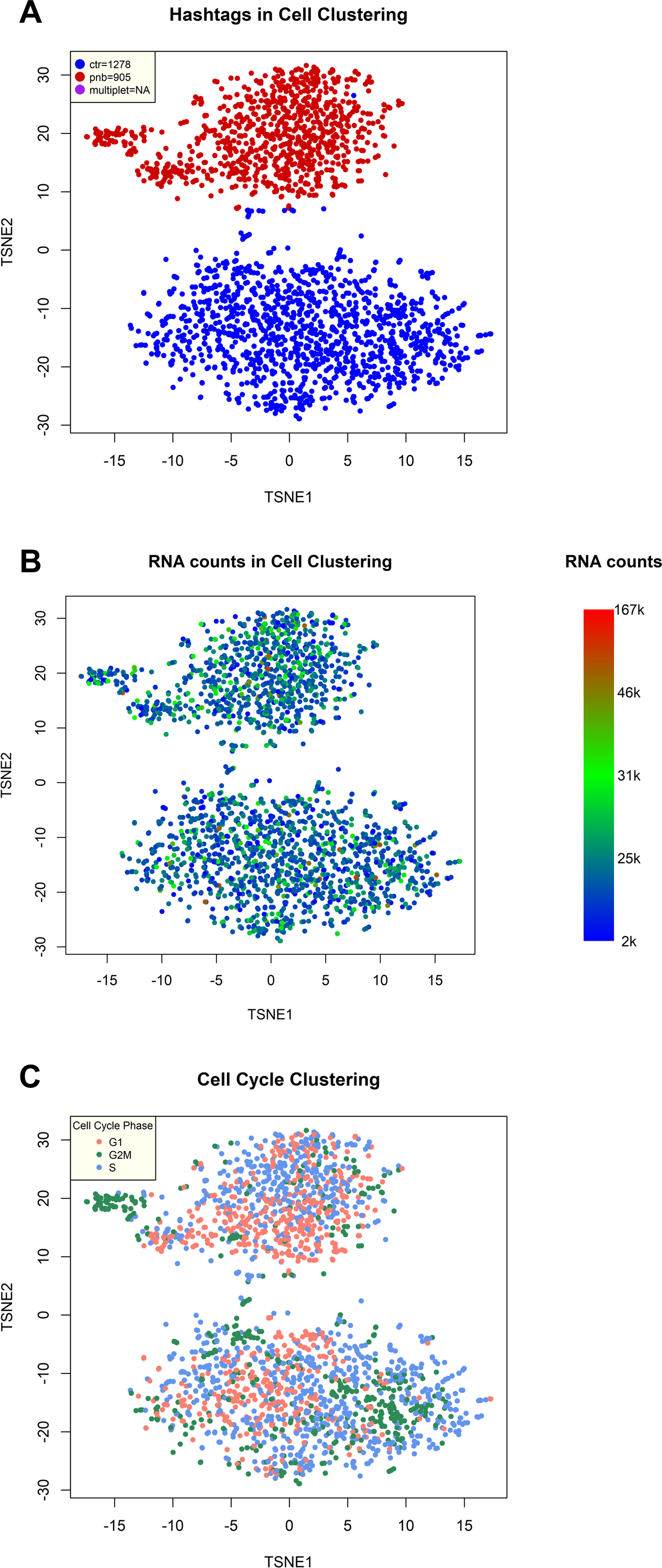

Supplement: Supplementary file 1 [file genes-13-02240-s001.zip › SupplementaryFigureS1.png]

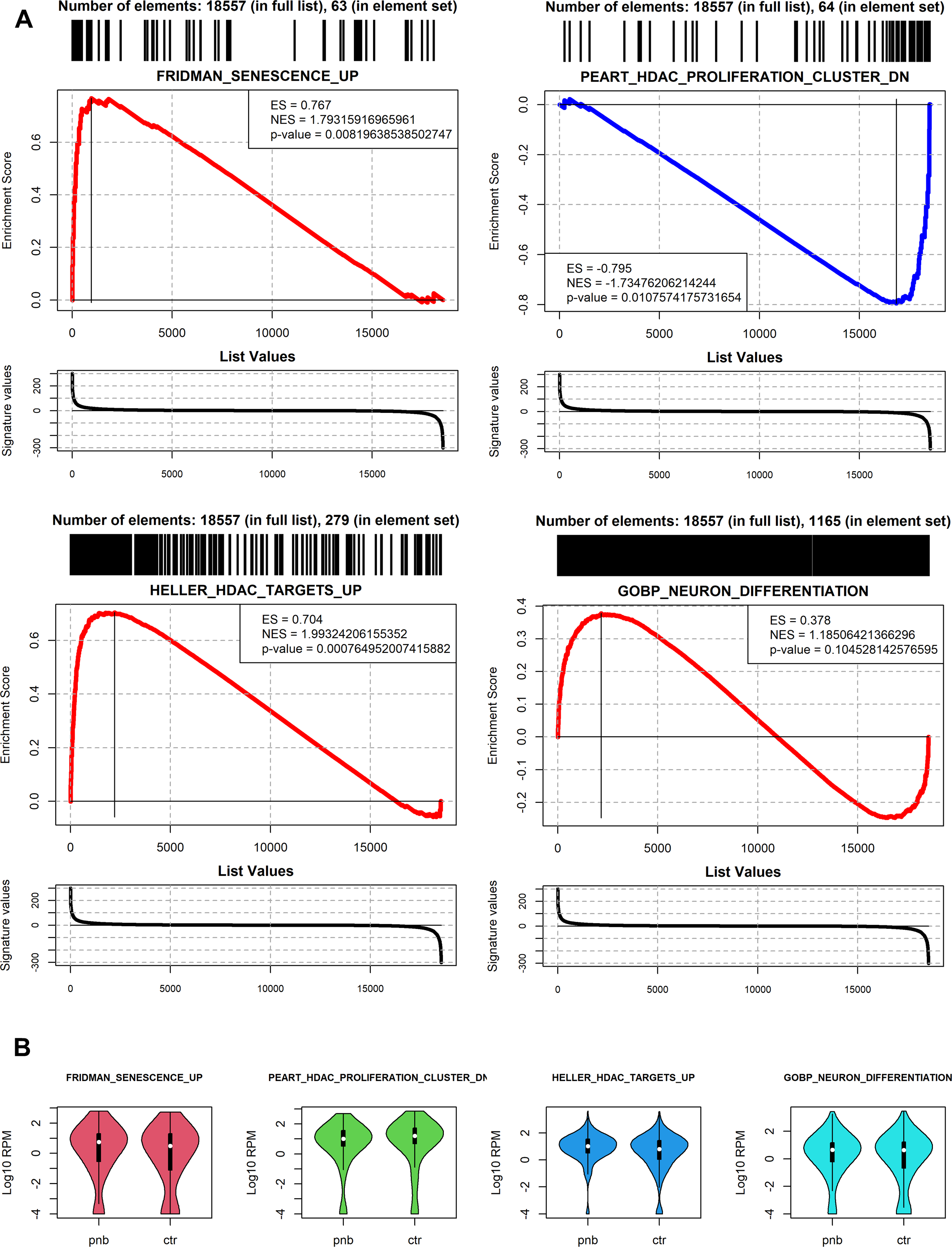

Supplement: Supplementary file 1 [file genes-13-02240-s001.zip › SupplementaryFigureS2.png]
